# Sharing the burden: The experiences of HIV psychiatrists delivering primary palliative care

**DOI:** 10.1017/S1478951525000124

**Published:** 2025-03-07

**Authors:** Sanam Bhatia, Maureen I. Ekwebelem, Chloe Nims, Catherine Riffin, M. Carrington Reid, Daniel Shalev

**Affiliations:** 1 Weill Cornell Medical College, New York, NY, USA; 2 Weill Cornell Division of Geriatrics and Palliative Medicine, Both Weill Cornell Medicine, New York, NY, USA; 3 Weill Cornell Department of Psychiatry, Both Weill Cornell Medicine, New York, NY, USA

**Keywords:** HIV psychiatry, palliative care, hospice, serious illness, consultation-liaison psychiatry

## Abstract

**Objectives:**

People living with HIV experience an elevated risk of serious medical illnesses as they age, but access palliative care (PC) at lower rates than individuals without HIV. HIV psychiatrists provide longitudinal psychosocial care to individuals living with HIV. As such, HIV psychiatrists can play an important role in providing PC to people living with HIV (PLWH). This qualitative study was conducted to explore the perspectives and experiences of HIV psychiatrists in addressing the PC of PLWH.

**Methods:**

We conducted semi-structured interviews with HIV psychiatrists. Data were analyzed using thematic analysis.

**Results:**

Nineteen HIV psychiatrists were interviewed. Three core themes with several subthemes were identified. These were: (1) lack of an operationalized role for HIV psychiatrists in supporting PC provision, (2) heterogeneity in engagement with PC among HIV psychiatrists, and (3) HIV psychiatrists have valuable skills to address patients’ PC needs but also face unique challenges in doing so.

**Significance of results:**

Overall, we found that there is significant heterogeneity in how HIV psychiatrists provide PC. Psychiatrists were interested in engagement with PC but felt their roles and scope were poorly defined. This study points to the possibility for greater integration of HIV psychiatrists in the provision of PC for patients with HIV through improvements in PC training for psychiatrists who work with patients with medical illness and through a more operationalized role and scope of practice in this domain of care.

## Introduction

Palliative care (PC) is holistic care that aims to relieve physical, psychosocial, and spiritual suffering and improve quality of life for patients with serious illnesses and their caregivers (What Are Palliative Care and Hospice Care? n.d.). PC and consultation-liaison psychiatry (CLP) have many areas of overlap, including supporting medical decision-making, addressing psychiatric symptoms in the context of serious illnesses, and exploring existential issues related to serious illnesses (Shalev et al. 2024). Domains in which there is less overlap include the greater focus on psychiatric diagnosis and treatment in CLP and the focus on addressing physical symptoms, advance care planning, and spiritual care in PC (Ferrell et al. 2018).

People living with HIV (PLWH) have high PC needs. Although the advent of highly active antiretroviral therapy (HAART) has made HIV a chronic, treatable illness, people living with HIV experience high rates of mental and medical comorbidities (Simone and Appelbaum 2008). As a result, PLWH die on average 8 years earlier than people without HIV (Marcus et al. 2016). Furthermore, by 2030, it is estimated that 70% of PLWH in the United States will be over 50 (Wing 2016). As PLWH age, they are at increased risk for functional limitations and disability (Morgan et al. 2012). Given the high burden of physical and psychiatric symptoms, frailty, medical complexity, and premature mortality among PLWH, the World Health Organization advocates for a “special focus on palliative care” for PLWH (Palliative care fact sheet n.d.). Unfortunately, evidence suggests that PLWH experience gaps in access to PC (Rhodes et al. 2016). For example, people with advanced cancer have a 30% lower chance of receiving PC if they have HIV (Islam et al. 2022).

One way of providing PC to populations without access is through primary PC (PC delivered by non-PC specialists) (Lupu et al. 2018; Guthrie 2023). Primary PC involves empowering non-PC specialists to integrate PC skills into their clinical care and has been identified as a key strategy to mitigate deficiencies in the PC workforce (Lupu et al. 2018; Guthrie 2023). HIV psychiatrists provide embedded behavioral health services within HIV clinical programs and function as pivotal members of HIV care teams. In providing longitudinal, biopsychosocial care, HIV psychiatrists are inherently providing care at the interface of CLP and PC. However, little primary PC scholarship or training focuses on psychiatrists, who may receive minimal, poorlyoperationalized exposure to nonpsychiatric components of PC (Fields et al. 2022; Shalev et al. 2020b). Furthermore, little is known about the experiences of psychiatrists addressing the PC needs of their patients. Some research has focused on geriatric psychiatrists specifically, suggesting that they value and utilize primary PC skills. However, there is no qualitative evidence regarding how psychiatrists may address the PC needs of their patients living with serious illness (Elhassan et al. 2023).

Given the high unmet PC needs of PLWH and the unique role of HIV psychiatrists (Cohen et al. 2019), this study aimed to explore the perspectives of and experiences of HIV psychiatrists in addressing the PC needs of their patients.

## Methods

### Study design

This was an exploratory-descriptive qualitative (Stanley 2014) study reported in accordance with the Consolidated Criteria for Reporting Qualitative Research (see supplement 1) (Tong et al. 2007). Semi-structured interviews were conducted with psychiatrists providing care to PLWH in the United States and Canada.

### Interview guide

The authors developed a semi-structured interview guide (supplement 2) leveraging data on the PC needs of PLWH (Harding 2018), PC psychiatry (Elhassan et al. 2023; Fields et al. 2022; Hurwitz et al. 2022; Shalev et al. 2020b), and the domains of PC (Ferrell et al. 2018). Interview guide content included (1) participants’ background in HIV psychiatry, (2) their involvement with specific domains of PC, and (3) barriers and facilitators to meeting PC needs. The interview guide was iteratively refined by a research team of men and women ranging from post-baccalaureate to full professors with expertise in geriatrics (MCR), PC (MCR, DS), consultation-liaison (DS) and HIV psychiatry (CN), and qualitative methods (CAR).

### Eligibility

English-speaking HIV psychiatrists practicing in the United States or Canada were eligible to participate in the study.

### Sampling and recruitment

We recruited a convenience sample of HIV psychiatrists via the senior author’s professional network, online directories, and snowball sampling (a method by which research participants recommend other potential participants) (Parker et al. 2019). Potential participants were invited to participate via email. They were not offered compensation for study participation. We continued recruitment until thematic saturation was achieved (Francis et al. 2010).

### Setting and data collection

Participants completed an electronic informed consent form. They did not receive the interview guide in advance. Interviews were conducted on Zoom between February and March 2023 by the primary author, a trained medical student. The interviewer introduced her role and the goals of the research: understanding the perspectives of HIV psychiatrists on delivering PC. Interviews were one-on-one and lasted between 20 and 50 minutes. Field notes were not taken. Zoom was used to record interviews and auto-generate transcripts, which were then refined and de-identified by the primary author.

### Data analysis

We used a thematic analysis approach facilitated by Atlas.Ti (v5.20.0–2016-11-28) using hybrid inductive–deductive coding (Braun and Clarke 2021). We developed an initial codebook based on the senior authors’ prior work at the interface of PC and psychiatry (Elhassan et al. 2023; Fields et al. 2022; Shalev et al. 2020a, 2020b). Then, SB and ME independently coded transcripts based on the preliminary codebook and identified new codes inductively. Iterative back-coding was used to code transcripts using the final codebook. Consensus meetings were held during this process to refine the codes and review coded transcripts. We considered saturation achieved after no new codes were generated in 5 interviews. Following coding, key themes were identified through discussion and data review. An audit trail was maintained throughout the coding process to keep track of analytic decisions. Participants did re-review transcripts or provide feedback on these findings.

### Ethics

The Weill Cornell Institutional Review Board approved this study.

## Results

### Study population

We invited 51 HIV psychiatrists to participate. Twenty-eight did not respond. Four declined due to time constraints or lack of expertise. Nineteen participants were interviewed (see Table 1). Eleven were men. All but one participant (from Canada) practiced in the United States, with most working in the Northeast. Participants’ clinical experience with HIV psychiatry ranged from a year to multiple decades. Seven participants completed some training in internal medicine, and several completed consultation-liaison (CL) fellowships (*n* = 7). Most of the participants practiced HIV psychiatry in the outpatient setting (*n* = 13). Most participants worked primarily with patients with HIV. Two study participants were colleagues of co-author CN, and co-authors CN and DS were professionally acquainted with several study participants through national CL psychiatry organizations.

**Table 1.** Participant characteristics

Gender	*N* (%)
Men	11 (58%)
Woman	8 (42%)
Early Career (≤5 years post-training)	3 (20%)
Location	
Northeast	13 (68%)
Southeast	2 (11%)
Mid-west	1 (5%)
West	2 (11%)
Canada	1 (5%)
Clinical post-residency training (some participants completed more than one)	
No formal fellowship	8 (42%)
Consultation-liaison	7 (37%)
Addiction	2 (11%)
Palliative care	1 (5%)
Child and adolescent	1 (5%)

### Thematic analysis

We uncovered 3 overarching themes and 7 sub-themes. See Tables 2–4 for illustrative quotes.

**Table 2.** Theme I

Theme I: There is a lack of guidance regarding the scope and roles of HIV psychiatrists in providing PC.	
*I-a: There are challenges in identifying patients who need PC.*	“More recently there have been patients who have had HIV for a very long time and have multiple other comorbidities – HIV not being the biggest one in that regard – who we’ve followed, especially when they’ve been in the hospital where we do talk more about death and dying especially. Someone who comes in maybe status post an MI, or status post, you know, the tenth fall. And trying to sort of try to think about, a little bit more about the goals of care and what are they hoping for in the expectation of their final years of life?” – PC006
*I-b: The scope of HIV psychiatrists in the context of PC is not clearly defined.*	“I’m not … I don’t directly prescribe for any [pain medications], cause there’s so many people involved now… our patients have their own HIV doctor, and then there’s the hospitalist … Sometimes, I will call things to the attention of others, but most of the time the patients themselves are making their pain needs known. Pain management is often involved. There are a lot of internal medicine people involved.” – PC003
	“When California first had the law where patients could ends their lives, if they’re not going to live beyond 6 months, we started to do those forms for them where they needed a psychiatrist to sign off on it, and we had a number of patients doing that, and where I got involved. But we haven’t done any of those in a long time, because I think it might be it’s being done by a different … Maybe it’s a palliative care service that’s doing that now? But they don’t come to me at least anymore for that.” – PC014
*I-c: HIV psychiatrists do not have a defined role delivering PC within the clinical team.*	“When my patients for psychiatry are inpatient, the team is gonna do what team is gonna do. And then they send them back to you. Right? They call you to get the history. What’s going on, but in terms of … it’s like if there’s drug-drug interactions, or sometimes EKG changes that some of our medications can cause.” – PC013
	“For a lot of these patients, I’m a relative newcomer in their care, as opposed to their infectious disease doc, for example, who they’ve maybe had for 20 years. They have a more established relationship with that person, even though it’s not a psychiatric treatment relationship. So they may feel more comfortable talking to that person about [death and dying].” – PC001

**Table 3.** Theme II

Theme	Quote
Theme II: There is heterogeneity in HIV psychiatrists’ contributions to PC.	
*II-a: There is heterogeneity in the ways in which HIV psychiatrists contribute to physical symptom management.*	”If a patient is depressed or anxious, and I know that they also have a pain syndrome, I’m more likely to start something with like an SNRI rather than an SSRI. I’m more likely to use something like Neurontin for anxiety, or Lamictal for depression that has a bipolar component trying to get some of the pain management pieces connected.” – PC012
	“I wasn’t the one prescribing fentanyl patches or anything like that. I didn’t do that, but from a psychological point of view I was often involved in keeping somebody company as they were dying.” – PC003
*II-b: HIV psychiatrists utilize both exploratory and goal-driven communication strategies about PC topics.*	“I frequently encourage my patients over 40 who don’t have an advanced directive on file to just fill one out, because it’s best to have one done long before you need it.” – PC008
	“We’ll have usually brief conversations about what that would mean and what kinds of decisions those would be, asking briefly about their general philosophy, [for example, if you] were severely injured, there was little chance of you being resuscitated and ever walking out of the hospital, what would you want to have done?” – PC008
	“I think, as a psychiatrist, I kind of know enough about the medical stuff that’s going on. So, I have a good sense of the end points to life. You know, whether it’s renal failure, whether it’s heart disease, whether it’s sort of more dementia, whether it’s more pulmonary. I’m not as good and kind of feel as comfortable about a lot of the medical details, and so I sometimes will, sometimes I’ll ask patients, what’s their understanding of these various things which helps me understand it a little bit better, because then it can help me phrase like, how I want to ask the internist about something. So, I think, being able to understand what the patient’s understanding of what their condition is important, and I feel like I’m not playing ignorant, because I’m kind of ignorant … about some of the things.” – PC006

**Table 4.** Theme III

Theme III: The experience of HIV psychiatrists in delivering PC is informed by their longitudinal relationships with patients and their liaison relationships with clinicians.	
*III-b: HIV psychiatrists accompany patients and their loved ones through serious illness.*	“As a psychiatrist, to be able to function in many ways, like some of the primary care doctors do where, you get to know people’s families and their spouses and their children. And now we’re seeing the whole … another generation pop up, of adults who were perinatally infected, and we actually knew their parents and watched them die. And now we’re keeping their kids alive, and their grandchildren alive. It’s really an unusual kind of career to have for a psychiatrist.” – PC003
	“I encouraged people to bring their children into the nursing home. There was a lot of confusion about, oh, my goodness, you know, kids shouldn’t see … Of course, they should! Kids should have an opportunity to deal with their parents at the end of life, and I encouraged everyone to have their kids brought in so not only could they play with them or talk with them or read to them, but if necessary, say goodbye and we did that. [Also] they never allow kids under a certain age into ICU. But well, we had little kids dressed up like little doctors or nurses with caps and gowns that were way too big, and, and got them into the intensive care unit.” – PC002
*III-c: HIV psychiatrists manage their – and their colleagues’ – difficulties with addressing death and dying.*	“I think internal medicine mainly would benefit from learning more about how to start conversations about the end of life and loss of autonomy and independence, because from my experience, it’s very challenging for them to have these conversations with patients. And sometimes they consult us, because, consciously or pre-consciously, they are not able to do [it] … They have a very reactive approach to illness, and everything is done very quickly. And these conversations usually require time, require you to sit down with the patient, understand who the patient is, what the patient’s values and goals are. And I think some primary teams find it difficult, and others just don’t like to do it.” – PC007
	“It totally is devastating to watch someone die who’s like under 30 or under 40, when you know it’s preventable.” – PC010
	“It often felt like I was treating the relationship between the physician and the patient or between the team and the patient. So, we would talk about what was going on, and sometimes [I] could get the patient to and the team – mainly the team – to turn the corner and say, let’s move to symptom management and patient focused care and palliative care. And that was really difficult, but really rewarding.” – PC010

#### Theme I: There is a lack of guidance regarding the scope and roles of HIV psychiatrists in providing PC

Participants noted that there was a lack of guidance in identifying patients who would benefit from PC interventions, understanding their scope of practice, and establishing their role on their teams vis-à-vis PC delivery.

##### Subtheme I-a: There are challenges in identifying patients who need PC

Participants explained that they do not universally discuss PC-related topics. Rather, they raised these topics with sicker patients. Participants emphasized that knowing which patients would benefit from PC – and when – is challenging. They highlighted situations in which they do not discuss PC, including with young and healthy patients with well-controlled HIV, with new patients, or with patients unable to manage such conversations. For example, one participant discussed the impact of patients’ decades-old grief related to HIV on their ability to engage in serious illness conversations:

To hear all of these stories about gay men living with HIV who had buried a hundred friends and lovers and former boyfriends in the span of a couple of years. That was 30 years ago, and I have a handful of patients who’ve gone through that, but that’s not on their minds these days. And I don’t see any need to go digging into old traumas (PC008).

Participants emphasized that decisions regarding which patients are appropriate for PC are complex now that HIV is a chronic illness. One explained that in the past, conversations about death and dying arose more frequently: “Life expectancy was not as long, always with fingers crossed that a new med would come out. That’s much less the case now so” (PC006). Another participant shared that the focus on such conversations in HIV psychiatry has changed with the changes in HIV care itself:

From a psychological point of view, I was often involved in keeping somebody company as they were dying. And so, we’re talking with people a lot about facing death and what it meant to them, and how they wanted to talk to their children, and what they wanted to leave behind for their children, and where they imagined they would be going, and those kinds of conversations were not particularly difficult because we were having them all the time … [currently] we don’t have a lot of patients that are really close to death. We just don’t. And, you know, obviously one shouldn’t wait until someone is close to death to have the conversations (PC003).

##### Subtheme I-b: The scope of HIV psychiatrists in the context of PC is not clearly defined

Participants varied in their perceived scope of practice regarding referral and primary PC. Few participants directly referred to PC, and only for psychiatry-related indications: “If I’m going to involve palliative care, it’s usually related to cognitive issues. Indirectly, I may hear about or make recommendations for people who are declining due to more physical issues. But the ID provider usually … manages that situation” (PC009). Participants noted that social workers are key in referring patients to key PC resources and helping patients complete advance directives.

Participants generally perceived that patients’ internists better served medical PC needs such as pain management and complex medical decision-making. Additionally, some participants conceptualized PC in opposition to HIV psychiatry. One shared, “I feel like, in general, the job of the psychiatrist is to relieve or improve mental suffering, and I’d be starting a discussion that would kind of create suffering, even if I might feel it’s like realistic” (PC002). Another said “when I talk to patients about patient care, I talk about function, quality of life and longevity. Those are my goals for patient care. Comfort’s not on the list till those things are not on the list” (PC015).

##### Subtheme I-c: HIV psychiatrists do not have a defined role delivering PC within the clinical team

Even when participants felt equipped to address PC issues with patients, many felt that team roles and dynamics complicated their involvement. Some described situations in which they noticed medical teams did not introduce PC-related conversations. For example, one participant described a seriously ill patient and said:

The whole discussion from the primary team and from the patient is all about recovery. It’s about getting better … he’s deteriorated in terms of his muscle strength. And he’s not able to walk anymore. In my mind, this seems like a good case for more extensive discussion about end-of-life wishes and thoughts about care (PC002).

However, the same participant also described feeling uncomfortable making suggestions to the primary team, saying, “I almost never have the approach of recommending that primary teams consult other services because I feel like that’s not my style … even if I might think that talking to palliative or other services might be helpful. I just stay in my lane” (PC002).

#### Theme II: There is heterogeneity in HIV psychiatrists’ contributions to PC

While nearly all participants contributed to meeting patients’ PC needs, their means differed. In part, participants noted that their variation in practice reflects the lack of standardization in case finding, scope of practice, and team roles. Specific elements of PC practice included conversations about the illness experience, conversations about death, dying, and existential concerns, advanced care planning, and physical symptom management. In general, participants were more involved in those domains that overlapped with CLP expertise than in other domains of PC.

##### Subtheme II-a: There is heterogeneity in how HIV psychiatrists contribute to physical symptom management

Participants described limited roles in physical symptom management. Some participants did not conceive of their role as focused on symptom management: “Things relating to pain management, psychiatry just doesn’t do. We’ll be engaged in the discussion. But we don’t write opioids. Really, the internist has taken charge of those” (PC006). While others described a range of roles at the interface of psychiatry and physical symptom management:

HIV causes fatigue, for example. Fatigue could be a symptom of depression, too. Right? So we had to say, is this coming from the, the … Medication could cause fatigue too. So is this coming from a medication? Is this is coming from an underlying psychiatric issue. Is it coming just part of the HIV? Same thing with insomnia, for example, it’s very common, right? Pain as well, you know. As you may know, people with anxiety sometimes tend to somaticize a lot, right? (PC005)

None of the participants felt that HIV psychiatry’s role was to be the driver of physical symptom management.

##### Subtheme II-b: HIV psychiatrists utilize both exploratory and goal-driven communication strategies about end-of-life care

Though most HIV psychiatrists described discussions about death and dying, these conversations were often focusing on the psychological consequences of death anxiety and coping with serious illnesses, but less so on physical symptoms:

[I’d say] tell me how you’re sleeping, and then, the patient would usually say, I’m not sleeping, and then I would say, well, what are the top three themes that keep you awake at night, and often death anxiety was in that. Thoughts about what if I don’t make it, or loss, or separation, or just anguish. And if it was okay, and I had rapport with the patient, I’d say, so when you think about death, what do you think about, which is the most insanely broad question you could ask (PC010).

Participants described these conversations as exploratory rather than goal-driven in nature. Many participants noted that engaging patients in these discussions about death was a helpful intervention. One participant recalled asking patients:

What do you think will happen when you die? And what do you think will happen after you die. Cause I found that for some patients that can be a very comforting way of kind of talking about some of the aspects of end-of-life care, … And I feel like, you know, being able to bring it into the room tends to do a lot more good than harm (PC006)

In contrast, some participants also described goal-directed conversations, particularly regarding advance directives. Some participants discussed encouraging the completion of advance directives: “I frequently encourage my patients over 40 who don’t have an advanced directive on file to just fill one out, because it’s best to have one done long before you need it” (PC008).

Other participants described working with patients actually to complete advance directives:

I want to know from all of my patients if they ended up in a car accident, then they’re on a respirator, or they were taken to the ER, what are their wishes? I really work with all of my patients to make an end-of-life will, a health care proxy, [to ensure] that the person who is going to make decisions for them knows what their wishes are (PC004).

#### Theme III: The experience of HIV psychiatrists in delivering PC is informed by their longitudinal relationships with patients and their liaison relationships with clinicians

##### Subtheme III-a: HIV psychiatrists accompany patients and their loved ones through serious illness

Participants described how their close relationship with patients impacted their approach to PC. One participant discussed how, in the 80s and 90s, “bonds established between providers and patients were enormously intense and special … [it is] very, very privileged, to be with somebody while they’re dying … often people had been abandoned by their families” (PC003). Because of the longitudinal nature of psychiatric treatment, participants spoke about experiencing their own illness and losses during a patient relationship: “they’ve seen me lose weight and even be impacted by my own health issues and sort of drag myself back up. They’ve seen an undulating life course for me, which I think is really kind of important” (PC012).

Participants felt called to advocate for their patients’ PC needs in the context of many inequities that PLWH experience: “Because these patients tend to have poor social support, they don’t have a lot of people to advocate for them, so we end up being the ones advocating for them, and I think that’s our major role in those [end-of-life] situations” (PC007).

Participants felt that the social component of PC was particularly important for HIV psychiatrists to address:

The hardest cases are our patients who are unbefriended or unrepresented [for whom] we can’t find anybody. We don’t really know enough about them to figure out what they would want and what they wouldn’t want. [But] we’ve had people who are friends, distant cousins, sometimes a distant child … Reconnecting people when they’ve been estranged for a very long time … can be really rewarding. (PC011).

In such examples, though the psychiatrist does not directly drive medical decision-making, their work of reconnection informs the identification of surrogate decision-makers and, thus, PC delivery.

##### Subtheme III-b: HIV psychiatrists manage their – and their colleagues’ – difficulties with addressing death and dying

Participants shared that though psychiatrists may struggle to manage their own emotions about death, they must also address the reactions of other clinicians. Participants recalled difficult moments with patients before the use of HAART:

Early on, one patient in particular … I remember I had tears running down my face and he was young, he was in his thirties and this was really the end of his life, and he had to move into a hospice at that point. But it backfired on me. He didn’t want to see me again after that. It was, too … He thought he was hurting me too much, and so that was hard. But I’ve tried to be stronger, but still able to share the time with them (PC014).

Another recalled how they occasionally remember patients who passed away from HIV/AIDS, saying,

I definitely was getting some flashbacks to internship year when I would meet someone … they’d be in late stage, they would be really fun, like wearing a jacket and using their IV pole as a microphone, and then, the following week, they were gone … sometimes I would get intrusive thoughts about really cool patients that I had lost, but effectively, had to work with death and the counter-transference, and I stayed in psychotherapy … and I talked through my own loss issues when I would lose someone (PC010).

One participant noticed psychiatric colleagues’ discomfort in discussing mortality and grief:

Every physician, but especially every psychiatrist, should work on feeling comfortable having these conversations. I’m always kind of struck by on our listserv, there will be people asking, you know, does anyone know a grief counselor I could send this person to? Or does anyone know somebody who could talk to this patient about the end of their lives (PC003).

Several participants noted that medical clinicians also demonstrate discomfort in managing patients at the end of life:

I work with internal medicine residents, a lot of whom don’t get a lot psychiatric experience and training, depending on the person, they’re very focused on … the medicine and let’s get him out of hospital … I feel pretty comfortable stepping back and saying, okay, let’s talk about our goals here … and that often opens conversations about palliative care. (PC009).

Several HIV psychiatrists commented on challenging cultural attitudes about mortality in medicine: “A lot of what our medical training teaches us, and our experiences in the hospital, is that death is never okay.” They described “managing death anxiety in providers … [and] really helping them be comfortable with their feelings too, because if they’re not comfortable with their feelings, patients can tell” (PC011).

## Discussion

This qualitative study highlights the important but heterogeneous role of HIV psychiatrists in providing primary PC. Despite a lack of clarity in role and scope of practice, HIV psychiatrists attend to patients’ PC needs in a variety of ways. Many of the interventions described by participants are at the interface of CLP and PC, including accompanying dying patients, supporting colleagues, supporting patients’ existential and psychological health, and advocating for disenfranchised patients. Few HIV psychiatrists were directly involved with the medical components of PC, such as physical symptom management. Our findings are consistent with previous research, which has demonstrated that psychiatrists engage in a range of PC practices (Elhassan et al. 2023), facilitated by unique key skills and experiences (Fairman and Irwin 2013), but often lack formalized training and structure (Fields et al. 2022; Shalev et al. 2020b).

Our study has several limitations. Principal among these are the significant overlaps between CLP and PC in psychological and emotional assessment of illness coping, exploration of health-related goals and preferences, and management of psychiatric symptoms in serious illness (Ferrell et al. 2018). There was ambiguity in many of our participants’ responses regarding whether they were practicing primary PC, CLP, or both. Additionally, while we recruited nationally, most of our participants practiced in the northeast and academic medical centers. Furthermore, the small number of HIV psychiatrists may have affected participants’ ability to speak honestly about their experiences due to anonymity concerns.

Our findings highlight the important role psychiatrists can play in meeting the PC needs of patients with dual medical and mental co-morbidities, while identifying barriers that limit this role. In particular, we identified a need for greater training opportunities for psychiatrists to learn key PC skills, as there are currently limited opportunities during residency and fellowship (Fields et al. 2022; Hurwitz et al. 2022; Shalev et al. 2020b). Optimizing this domain of CLP training depends on better elucidating shared skillsets between CLP and PC and understanding high-yield additional skills from which CLPs may benefit. Future studies should assess the experiences of psychiatrists working with patients with other illnesses, such as cancer or cardiovascular disease, as well as the specific PC responsibilities of psychiatrists across settings.

## Conclusion

In this qualitative study, we found that HIV psychiatrists address the emotional and psychological components of PC through discussions with patients about death and dying, therapeutic accompaniment, encouragement for completion of advance directives, and addressing psychiatric symptoms of serious illness. Many of these interventions were situated at the interface of CLP and PC, and few participants addressed PC’s more “medical” components, such as physical symptom management. Future training and models of care should focus on supporting the role of HIV psychiatrists in meeting emotional and psychological PC needs while exploring the potential to expand their role to other forms of primary PC.

## Supporting information

Bhatia et al. supplementary material 1 Bhatia et al. supplementary material

Bhatia et al. supplementary material 2 Bhatia et al. supplementary material

## Supplementary material

The supplementary material for this article can be found at https://doi.org/10.1017/S1478951525000124.
