# Supplementary material for: Sharing the burden: The experiences of HIV psychiatrists delivering primary palliative care
Source: Palliat Support Care. 2025 Mar 7;23:e73. doi: 10.1017/S1478951525000124 (PMC11893077; doi:10.1017/S1478951525000124)
Supplement: Bhatia et al. supplementary material 2 — Bhatia et al. supplementary material [file S1478951525000124sup002.docx]

**Supplement 1: Interview Guide**

**HIV and Palliative Care Interview Guide**

*Thank you for agreeing to participate in this study. While HIV is no longer an acutely life-threatening illness for many patients, the World Health Organization recommends that palliative care be incorporated into every level of management for people living with HIV. Thus, we hope to better understand the role that HIV psychiatrists in particular play in the provision of palliative care for patients with HIV. There are no right or wrong answers; we want to learn about your perspectives and experiences.*

*Participation in this study is voluntary. This interview will take about 45 minutes depending on our conversation. With your permission, I will be recording the interview so that I don’t miss your comments. Your responses will be confidential; nothing you share will be linked to you specifically. You can decline to answer any question or stop the interview at any time. Do you have any questions?*

*I will now turn on the recorder.*

**Background on HIV psychiatry**

*To start off, we would like to learn about* your *experience and* *work as an HIV psychiatrist****.***

- Did you complete post-residency training?
  - Such as a fellowship?
  - Or any other specialized training programs?
- Can you describe the range of services provided in your clinical setting?
  - Does your clinic provide psychotherapy?
  - Medication management?
  - Substance use treatment?
- Can you describe your clinical role?
  - In what settings do you work with patients?
    - Outpatient clinic
    - Inpatient/hospital
    - Home visits
- Can you describe your patient population?
  - For example, what is the age range of your patients?
    - Can you tell me what proportion of your patients you would estimate are above the age of 50?
  - Can you describe the socioeconomic status distribution of your patient population?
  - Can you describe the gender and sexual orientation breakdown of your patient population?
- How long have you been practicing as an HIV psychiatrist?
  - During your career as an HIV psychiatrist, how has your patient population changed, if at all?
    - Have you noticed a change in demographics, particularly age?
    - How about a change in how acutely ill they are?
- Can you tell me about the primary psychiatric health needs of your patient population?

*Next, we would like to learn about some specific aspects of your clinical practice, including whether you provide any forms of palliative care to patients.*

**Discussing end-of-life care**

- What proportion of your patients would you estimate have comorbid, potentially life-threatening illnesses, such as poorly controlled HIV, cancer, advanced CHF, advanced COPD or multiple chronic conditions?
- What proportion of your patients would you estimate have a prognosis under five years?
  - Under one year?
- Can you tell me about your familiarity with palliative care?
  - *Palliative care is specialized medical care for people living with a serious illness, such as cancer, heart failure, or HIV. It involves addressing the challenges associated with these illnesses, whether physical, psychological, social, or spiritual. It can also involve establishing goals of care that are consistent with the patient’s values and preferences.*
- Given your understanding of palliative care, as well as this definition, what do you perceive as the most important aspects of palliative care for your patients?
- Do your patients have a high physical symptom burden, including pain, dyspnea, nausea, etc?
  - Who typically manages these symptoms?
  - Do you evaluate or manage these symptoms in anyway? If yes, how so?
- What proportion of your patients would you estimate have completed advance care planning (ex. health care proxy, living wills, do not resuscitate orders, etc.)?
  - In general, who guides patients through the completion of these documents?
- In the context of your clinical practice, do you think of yourself as providing any aspects of palliative care for your patients?
  - Do you find yourself assisting with pain management?
  - Do you or other members of your team assist with the management of other symptoms like fatigue, nausea, dyspnea, etc.?
  - What do conversations surrounding your patients care preferences or “goals of care” look like?
    - Do you ever talk about what would happen if your patients were to become medically sicker?
    - Do you ever talk about what level of care your patients would prefer in this situation?
    - Do patients ever discuss whether they would want any loved ones involved in these decisions?
    - What do these discussions look like?
- How do discussions about mortality and/or death and dying factor into your practice?
  - Is this something that your patients wish to discuss?
  - Is this something that you make a point of bringing up with patients?
  - Who are the typical patients with whom you have these conversations?
    - Do they tend to have multiple comorbidities?
    - Do these patients have limited prognosis (less than one year, less than five years)?
    - Do they tend to be older?
- How do you interact with patient’s loved ones, including friends, family, or caregivers, when engaging in goals of cares discussions/advanced care planning?
  - How are patients’ family/friends/caregivers involved in these conversations?
- How do discussions relating to patients’ spiritual beliefs arise when discussing care preferences or goals of care?
  - Is this something that your patients wish to discuss?
  - Is this something that you make a point of bringing up with patients?
  - How do you navigate these conversations?
- Do you have patients who enroll in hospice care?
  - Can you tell me about the role might you play if or when that happens?
- When engaging in the aforementioned forms of care, including pain/symptom management, goals of care discussions and advance care planning, how do you collaborate with your team members, if at all?
  - What about providers from other teams or specialties (geriatrics, palliative care, primary care, etc.)?
  - What about chaplains?
- What are some challenges in providing palliative care to patients with mental health comorbidities and HIV?
  - Do you face time constraints?
  - Are patients hesitant to discuss death and dying?
  - What challenges do you face when providing palliative care to with limited prognosis?
- Are there ways that your training has prepared you to work with patients with limited prognosis?
  - Are there ways in which your training did not prepare you?
  - Is there one thing that would have been helpful if it had been included in your training?

**Further Information**

- Is there anything else that we have not touched upon and that you would like to share with us?
- Do you have any questions for us?

*Thank you for taking the time to speak with us.*
